# Recent Progress on the Fabrication and Properties of Silver Nanowire-Based Transparent Electrodes

**DOI:** 10.3390/nano8080628

**Published:** 2018-08-18

**Authors:** Renyun Zhang, Magnus Engholm

**Affiliations:** 1 Department of Natural Sciences, Mid Sweden University, SE-85170 Sundsvall, Sweden; 2 Department of Electronics Design, Mid Sweden University, SE-85170 Sundsvall, Sweden; magnus.engholm@miun.se

**Keywords:** transparent electrodes, silver nanowires, mechanical stabilities, chemical stabilities, thermal stabilities, optical properties

## Abstract

Transparent electrodes (TEs) made of metallic nanowires, such as Ag, Au, Cu, and Ni, are attracting increasing attention for several reasons: (1) they can act as a substitute for tin oxide-based TEs such as indium-tin oxide (ITO) and fluorine-doped tin oxide (FTO); (2) various methods exist for fabricating such TEs such as filtration, spraying, and Meyer bar coating; (3) greater compatibility with different substrates can be achieved due to the variety of fabrication methods; and (4) extra functions in addition to serving as electrodes, such as catalytic abilities, can be obtained due to the metals of which the TEs are composed. There are a large number of applications for TEs, ranging from electronics and sensors to biomedical devices. This short review is a summary of recent progress, mainly over the past five years, on silver nanowire-based TEs. The focus of the review is on theory development, mechanical, chemical, and thermal stability as well as optical properties. The many applications of TEs are outside the scope of this review.

## 1. Introduction

Transparent electrodes (TEs) are essential components in many optoelectronic applications such as solar cells, touch screen displays, and film heaters [1]. The materials used for making these electrodes are mainly transparent conductive oxides (TCOs) such as indium-tin oxide (ITO), fluorine-doped tin oxide (FTO), and doped zinc oxide. However, the low availability of these elements and the high demands imposed by the fabrication conditions lead to high prices for these TEs. In addition, the brittleness of the materials limits their application in new types of flexible electronics such as wearable sensors.

Therefore, new materials, such as conductive polymers, carbon nanotubes, graphene, ultra-thin metal films, and metallic nanowires, have been adopted for fabricating TEs. Despite the wide choice of materials, few of these can fulfill the requirements of industrial production (transmittance > 90% and sheet resistance < 100 Ω/sq) [2]. Research has shown that metallic nanowires can fulfill these requirements. Thus, increasing efforts have been made in this area, such as developing new methods for synthesizing nanowires, new deposition methods, and new post-deposition procedures.

Ag nanowires are the most studied material for making metallic nanowire-based TEs. One reason is that the methods for synthesizing silver nanowires are relatively simple and the chemical stability of silver is better than other metals such as copper. When compared to other nanowires such as carbon nanotubes, the advantage of silver nanowires is that they can create networks having better electrical conductivity. The most common route is to synthesize Ag nanowires followed by deposition on different substrates using methods such as electrostatic spraying [3,4], doctor blading [5], electroless deposition [6], Meyer bar coating [7], ink jet printing [8], and spin coating [9].

Results from studies before 2013 showed that bare Ag nanowires can easily be deposited on different substrates by using the techniques mentioned above, and the length to diameter ratio of the nanowire determines whether the nanowires have the potential to achieve the requirements for industrial production (transmittance > 90% and sheet resistance < 100 Ω/sq) [2].

Recent trends have been to enhance the electrical stability, flexibility, and chemical and thermal stabilities, to tune the optical properties and to apply these TEs in different optoelectronic applications. Recent reviews, see e.g., Sannicolo et al. or Xue et al. [10,11] have summarized the advantages of nanowire-based TEs and their applications in flexible electronics and optoelectronics.

This short review summarizes the main advances in silver nanowire-based TEs that have been made in the past five years. The review focuses on theory development, mechanical and chemical stability, and tuning of the optical properties.

## 2. Theoretical Approaches

### 2.1. Transparency and Conductivity

Transparency and conductivity are the two most important parameters for characterizing TEs. For a bulk-like film such as ITO, the transparency, which is usually quantitatively expressed as the transmittance (*T*), is related to the sheet resistance (*R*_S_) of the film [12]:

(1) $$T=e^{-\alpha /\sigma_{\mathrm{DC},B}R_{s}},$$

where α is the absorption coefficient and σ_DC,B_ is the bulk DC conductivity of the film.

Metallic nanowire-based TEs have different mechanisms for their transmittance, based on the free space [12] among the nanowires instead of the absorption coefficient of the material. This means that the density of the nanowires determines the transparency of the TEs. Depending on the density of the nanowires, the transparent films, represented by the relation between the transmittance and the sheet resistance, are found to be either bulk-like or percolative.

In the bulk-like regime, the transmittance can be expressed as:

(2) $$T={\left(1+\frac{Z_{0}}{2}\sigma_{\mathrm{Op}}t\right)}^{-2},$$

where $\sigma_{\mathrm{Op}}$ is the optical conductivity ($\sigma_{\mathrm{Op}}$ ≈ α/*Z*_0_) and *Z*_0_ is the impedance of free space (377 Ω) [13]. In percolation theory [14], the conductivity ($\sigma_{\mathrm{DC}}$) of the nanowire film is non-linearly related to the difference between the density of the nanowires per unit area, *n*, and the percolation threshold, *n_c_:*

(3) $$\sigma_{\mathrm{DC}}\propto {\left(n-n_{c}\right)}^{m},$$

*n_c_*, also called the percolation threshold, ref. [14] is the value at which the network has a percolation probability of 1/2, and the exponent *m* has been found, by Monte Carlo simulation, to be 4/3 [15]. The value of *n_c_* is determined by the length of the nanowires (*L*_NW_) using the equation [14]:

(4) $$n_{c}=5.63726/L_{\mathrm{NW}}{}^{2},$$

By combining Equations (2) and (3), one can predict the relation between the transmittance and the sheet resistance using an equation suggested by Grüner and co-workers [16,17]:

(5) $$T={\left(1+\frac{Z_{0}}{2R_{s}}\frac{\sigma_{\mathrm{Op}}}{\sigma_{\mathrm{DC}}}\right)}^{-2},$$

**Figure 1:**
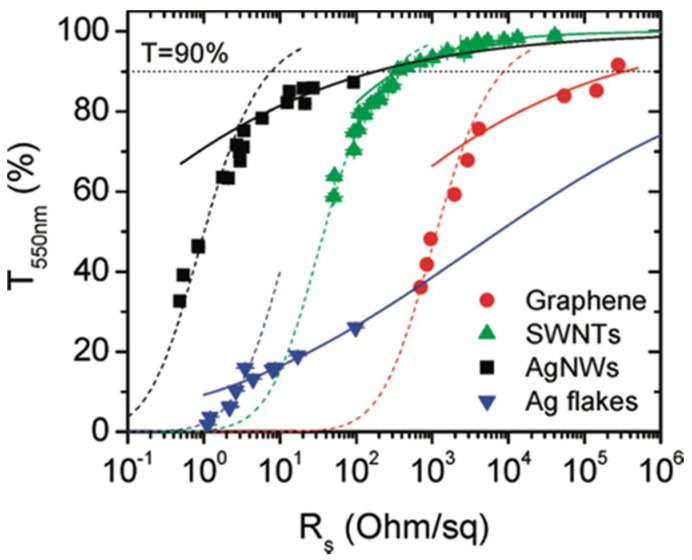
Transmittance (550 nm) plotted as a function of sheet resistance for thin films prepared from four nanostructured materials: graphene, single-walled carbon nanotubes, silver nanowires, and silver flakes. The dashed lines represent fits to the bulk regime using Equation (5), while the solid lines represent fits to the percolative regime using Equation (9). Reprinted from [12], with permission from American Chemical Society, 2010.

The experimental data from different materials with transmittance values from 0 to critical values, depending on the structure of the materials, can be fit using Equation (5) (Figure 1). Beyond the critical values, the data deviate from the fitting curves, and percolation theory should be applied.

The conductivity, $\sigma_{\mathrm{DC}}$ of a nanowire network film can also be expressed using the thickness of the films [15]:

(6) $$\sigma_{\mathrm{DC}}\propto {\left(t-t_{c}\right)}^{n},$$

where *t*_c_ is the threshold thickness and *n* is the percolation exponent. For a network with a conductivity high enough for industrial production, *t* is always greater than *t*_c_ [12]. Based on this, Coleman and co-workers [12] have further defined the relation between $\sigma_{\mathrm{DC}}$ and $\sigma_{\mathrm{DC},B}$ using the equation:

(7) $$\sigma_{\mathrm{DC}}=\sigma_{\mathrm{DC},B}{\left(\frac{t}{t_{\min}}\right)}^{n},$$

where *t*_min_ is the thickness of the nanowire network film that has $\sigma_{\mathrm{DC}}$ equal to $\sigma_{\mathrm{DC},B}$, usually 2.33 times the diameter of the nanowires.

Coleman et al. [12] have further used *n* to define a percolation Figure of Merit Π, that defines the relation between the transmittance *T* and the sheet resistance *R*_S_ of a TE:

(8) $$\Pi =2{\left[\frac{\frac{\sigma_{\mathrm{DC},B}}{\sigma_{\mathrm{Op}}}}{{\left(Z_{0}t_{\min}\sigma_{\mathrm{Op}}\right)}^{n}}\right]}^{\frac{1}{n+1}},$$

(9) $$T={\left[1+\frac{1}{\Pi }{\left(\frac{Z_{0}}{R_{s}}\right)}^{\frac{1}{n+1}}\right]}^{-2}.$$

By fitting the experimental data (Figure 1) with Equation (9), it is easy to get the values of Π and *n*. Π is a dimensionless number that reflect the values of the sheet resistance and the transmittance. The value of *n* reflects the junction resistance in the nanowire networks, where a small value indicates less junction resistance.

The fitting results in Figure 1 indicate that Equations (5) and (9) fit very well at the regime where it applies. Such fitting is of great importance to predict the possibility of the method to produce TEs that meet the requirements.

### 2.2. Transmission and Film Parameters

Mutiso and co-workers [18] developed another method to simulate nanowire-based networks. In their work, the sheet resistance is calculated based on the effective contact resistance (*R*_c_effective_) between the nanowires. They use the area fraction (*AF*) to define the density of the nanowires and, subsequently, the transmittance using the empirical equation [18]:

(10) $$T\%=100-a_{1}AF,$$

where *a*_1_ is a fitting parameter that accounts for the diameter and wavelength-dependent optical properties of the nanowires.

A plot of the transmittance and the sheet resistance shows a perfect fit using this method in both the bulk-like and percolative regimes. However, the ratio between the lengths and diameters must be given in order to perform the simulation.

Recently, Khanarian and co-workers [19] further developed the Mie light scattering theory of spheres and applied it to nanowires to predict both the transmission and the haze.

The transmission of a Ag-nanowire film with thickness *d* is given by:

(11) $$T=e^{-n_{v}C_{\mathrm{ext}}d},$$

where *C*_ext_ is the extinction coefficient of the nanowires given by Mie theory [20] and *n_v_* is the number of nanowires per volume, which is related to the volume fraction $\Phi_{v}$, the diameter *D*, and the length *L* by the equation below.

(12) $$n_{v}=\frac{\Phi_{v}}{L\pi \left(\frac{D^{2}}{4}\right)},$$

For TEs made of Ag nanowires with a thickness less than 100 nm, the transmission has been found to be better represented by:

(13) $$T=e^{-n_{s}C_{\mathrm{ext}}},$$

where *n_s_* is the number of nanowires per unit area.

This approach predicts the relationship between the transmittance of the TEs and the surface fraction ($\Phi_{s}$) of silver nanowires with respect to different diameters since *n_s_* can be presented as:

(14) $$n_{s}=\frac{\Phi_{s}}{DL}$$

### 2.3. Haze

Haze refers to the amount of light that is subject to wide scattering when passing through nanowire films. It is an important parameter for describing the optical properties of nanowire-based TEs. However, theoretical approaches that predict haze are limited.

**Figure 2:**
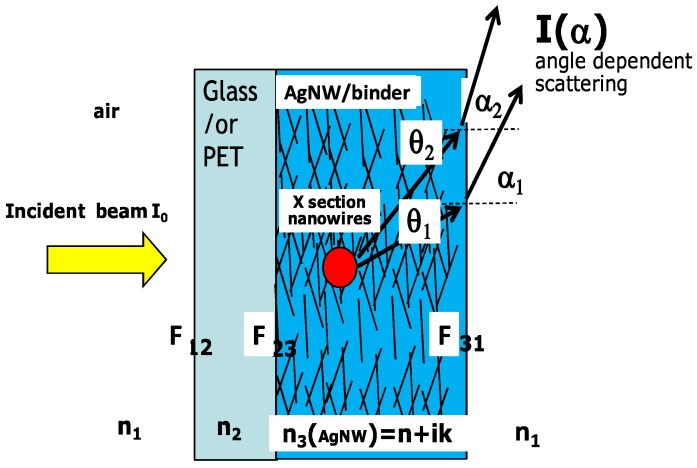
Scattering geometry of nanowires in films. Reprinted from [19], with the permission from AIP Publishing, 2013.

Khanarian and co-workers [19] extended the derivation provided by Willmouth [21] to derive an expression for haze in Ag nanowire films. The geometry of the scattered light passing through a Ag nanowire film is schematically presented in Figure 2. They further assumed that the Fresnel transmission terms [21] could be approximated by their average values at each optical interface. Then, an expression to predict the haze for nanowires was derived as follows:

(15) $$H=\frac{F_{12}F_{23}\frac{C_{2.5}^{90}}{C_{\mathrm{ext}}}\left(1-e^{-\Phi_{s}Q_{\mathrm{ext}}}\right)}{F_{12}F_{23}F_{31}e^{-\Phi_{s}Q_{\mathrm{ext}}}+F_{12}F_{23}\frac{C_{0}^{90}}{C_{\mathrm{ext}}}\left(1-e^{-\Phi_{s}Q_{\mathrm{ext}}}\right)},$$

where *F_ij_* are the Fresnel transmission terms, *Q*_ext_ is the scattering efficiency, $C_{m}^{n}$ is the scattering efficiency between angles *m* and *n*, and $\Phi_{s}$ is the surface fraction of the nanowires.

**Figure 3:**
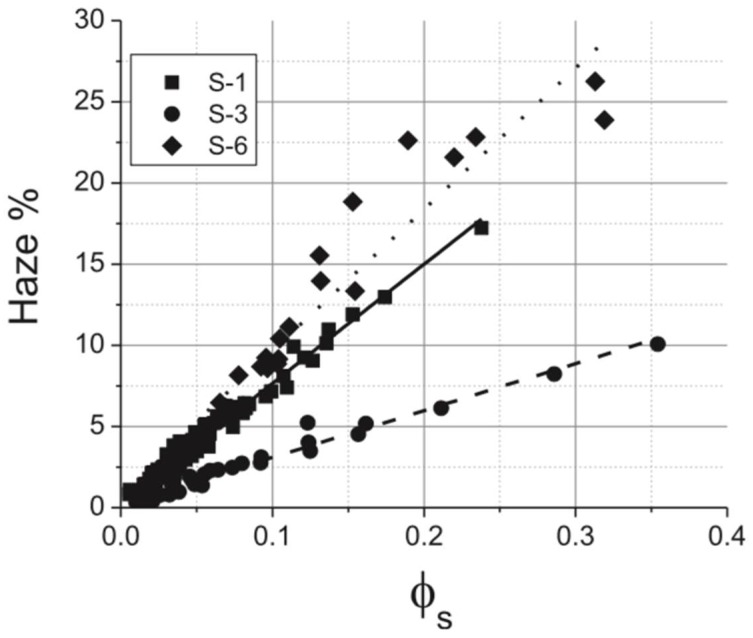
Optical haze versus silver area coverage /s for nanowires S-1 (100 nm), S-3 (56 nm), and S-6 (153 nm). Calculated curves are shown as (_____, S-1), (- - - - , S-3) and (........., S-6). Reprinted from [19], with permission from AIP Publishing, 2013.

The haze has a linear relationship with $\Phi_{s}$ (Figure 3) and increases significantly with the diameters of the silver nanowires. Such a phenomenon has been proved experimentally, which also agrees with Equation (15).

It follows from Figure 3 that the haze is strongly dependent on the diameter of the Ag nanowires. Hence, for applications that require a low haze (e.g. displays, *H* < 1%), it is found possible only for diameters less than ~50 nm.

Preston et al. [22] performed simulations and experiments on Ag nanowires and also came to the same conclusion. TEs with smaller Ag nanowire diameters have a lower haze and exhibit a better performance.

## 3. Experimental Approaches

### 3.1. Electrical Stability

Electrical stability [23] is one of the most important factors that needs to be considered to evaluate a silver nanowire based TE. It has been reported that silver nanowire networks break down due to electrical stress. Such a phenomenon could be due to electromigration [24] or elevated temperature [25] caused by Joule heating [26,27]. The joule heating occurs at the contact points of the silver nanowires, where a high contact resistance exists [28]. High current concentrates at the junction [27], leading to high current density with resulting Joule heating. The local temperature at the junction caused by Joule heating could reach 300 °C which leads to the melting of silver due to Plateau–Rayleigh instability [29]. The temperature could even go high enough to evaporate the silver [30]. Electromigration of silver happens when the nanowires suffer high current density. Under such a condition, the vacancies in the nanowires can sweep from anode to cathode or from cathode to anode [24], which creates a vacancy concentration gradient that results in a stress gradient. When the local stress gradient reaches a certain point, it will undergo an avalanche-like break down.

Attempts to increase the electrical stability of silver nanowire TEs include chemical treatment [31], electrical welding [32], laser sintering [33] etc. All these methods aim to reduce the resistance at junctions, which will reduce the local current density and thus reduce the Joule effect. However, such processed TEs have been shown not to fulfill the requirements for devices such as solar cells, as failure of the TEs have been observed under typical operational currents of solar cells [25].

An alternative way to increase the electrical stability is to coat the TEs with a thin layer of other materials such as ZnO [26] and poly(3,4-ethylene dioxythiophene):poly(styrene sulfonate) (PEDOT:PSS) [34]. The coating of such materials could delay the atomic surface diffusion of silver, since the atoms must diffuse through the coating layers before the deterioration of the junction can begin to occur [26].

### 3.2. Mechanical Stability

#### 3.2.1. Flexibility

Flexibility is a crucial factor for TEs, as it is of great importance for their application in soft electronics, e.g., as wearable sensors. Flexibility is a factor that combines the mechanical and electrical stability of a TE and is usually characterized by plotting the electrical conductivity versus the cycles of mechanical bending. Several protocols have been developed for enhancing the mechanical flexibility of TEs, such as welding or the deposition of extra material.

Welding is one of the more effective ways to enhance the flexibility of TEs while retaining their electrical properties. The welding procedure improves the contacts between the nanowires so that the nanowire network can withstand higher mechanical stress. Thermal annealing, light irradiation, mechanical pressing, plasma treatment, extra coating, cold welding, and chemical treatment are methods that have been used for welding Ag nanowires.

Hwang and co-workers demonstrated the welding of Ag nanowires by post-annealing at 180 °C for 25 min [4], thereby forming fused-in junctions among the nanowires. This welding procedure can significantly enhance the flexibility of TEs, while retaining their electrical resistance after 500,000 cycles with a strain of 1%.

The welding of Ag nanowires with intense light is also an effective method for enhancing the flexibility of TEs. Kou et al. [1] reported the welding of Ag nanowires using simulated sunlight (0.1 W/cm^2^). The welded TEs were shown to achieve the same flexibility as those welded at 200 °C. A 1.8 cm × 1.8 cm sized TE was found to maintain its electrical resistance after 500 cycles of bending to a minimum radius of curvature of 0.15 cm. In addition to simulated sunlight, flashlight irradiation has also been used to weld Ag nanowires. Li et al. [35] used flashlight irradiation with an energy of 4.6 J/cm^2^ per pulse to weld Ag nanowires on paper substrates. Hwan and co-workers [36] used a higher power density of 10.3 J/cm^2^ to weld Ag nanowires with an average diameter of 35 nm. High power lamps have also been used to weld Ag nanowires [37], where 10 to 120 seconds of irradiation under a tungsten-halogen lamp with a power density of 30 W/cm^2^ can weld the junctions between the nanowires.

Plasma treatment has also been found to cause the welding of Ag nanowires. Zhu and co-workers [38] treated Ag nanowire films with 75 W room-temperature plasma at room temperature. The treated film maintained its electrical resistance after 10,000 bending cycles at a frequency of 2 Hz.

Welding can also be done by post-deposition of 50 nm fluorine-doped ZnO (FZO) using a laser deposition method [9]. Using this method, no change in electrical resistance was observed after 1000 bending cycles, although the strain of the bending was not reported. Cheong et al. [39] welded Ag nanowires by depositing 30 nm and 50 nm indium tin oxide (ITO) using a DC magnetron sputtering system. The welded films maintained their electrical resistance during 10,000 bending cycles.

Capillary-force-induced cold welding [40] is a new method to create self-limited welding of the wire–wire junctions. These processes can be performed simply by applying moisture to the Ag nanowire films. However, this cold welding has not been fully studied, and the electrical resistance decreased at a bending radius of 1.5 mm.

**Figure 4:**
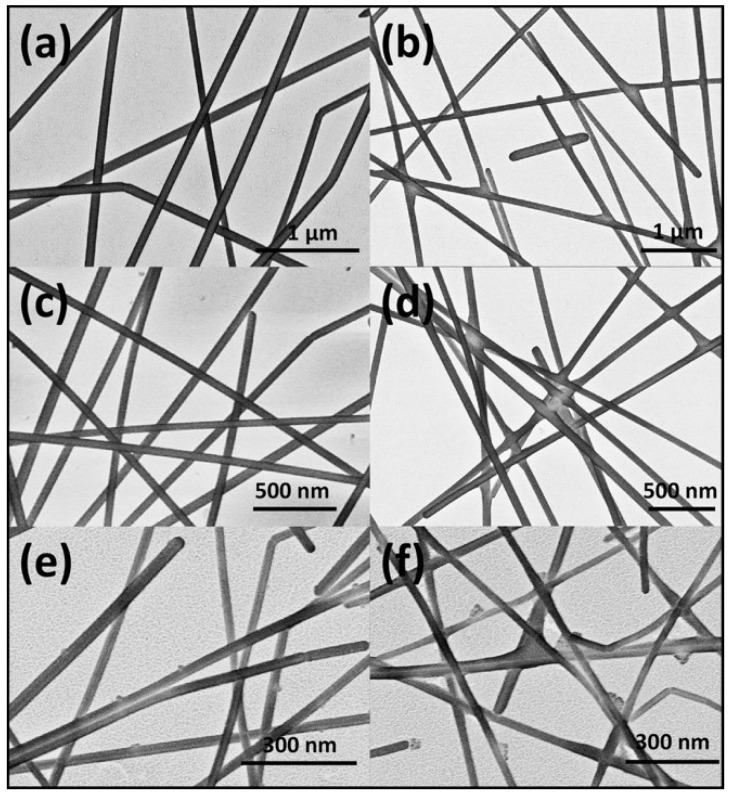
Scanning electron microscopy (SEM) images of transparent electrodes fabricated from (**a**,**b**) 100 nm, (**c**,**d**) 60 nm, and (**e**,**f**) 20 nm silver nanowires before and after chemical treatment. Reprinted from [31], with permission from John Wiley & Sons, Inc., 2015.

Chemical methods have also been reported to weld Ag nanowire films [31]. These methods are used for the deposition of Ag atoms at the junctions of the nanowires (Figure 4) because of the high local chemical potential of the concave surface [41]. The process can be easily performed by dipping the films in silver-containing solutions such as silver–ammonia [42].

#### 3.2.2. Adhesion

Adhesion is another important parameter for the mechanical stability of TEs. The Ag nanowire films are not self-supporting, and thus, supporting substrates are required to hold the films. However, bare Ag nanowire films do not adhere well to most substrates, and additional procedures are required to enhance the adhesion.

There are two general routes for enhancing the adhesion between Ag nanowires and substrates. The first one directly increases the adhesion by using extra processes; the second one requires the addition of other materials.

Spontaneous heating of the Ag nanowires and the substrate can lead to stronger contact between the nanowires and the substrate, resulting in stronger adhesion. Lee and co-workers [43] laminated Ag nanowires on a polyethylene terephthalate (PET) substrate at 120 °C to enhance the adhesion. Alternatively, other research groups used intense-pulse-light (IPL) to increase the adhesion between the nanowires and a substrate [44,45]. The IPL method can heat the nanowires and the substrate to a high temperature for a short period, thus increasing the contact between the materials.

In addition to heating, forces added to the films can increase the adhesion. A strong conformal pressure applied to the Ag nanowires can improve their adhesion to PET [46]. Liu and co-workers [31] noted that the capillary force produced by moisture on a Ag nanowire film can pull the nanowires to the substrate.

Although the methods mentioned above have been shown to increase the adhesion, they are less popular than those assisted by extra coating or by compositing the nanowire with substrate materials.

**Figure 5:**
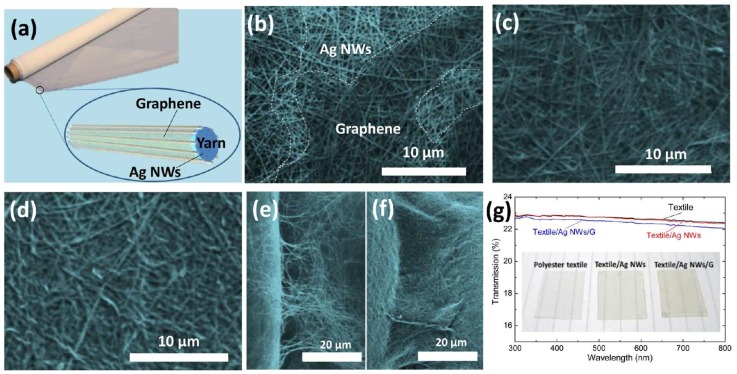
(**a**) Schematic diagram of an e-textile with a polyester/Ag nanowire (NW)/graphene core–shell structure. (**b**–**d**) SEM images of polyester/Ag NW/graphene samples with different numbers of graphene-coating cycles: (**b**) one cycle, (**c**) two cycles, and (**d**) three cycles. SEM images of the fiber cross-linked regions (**e**) before graphene coating and (**f**) after graphene coating. (**g**) Visible-light transmittance of the textile, the textile/Ag NW, and the textile/Ag NW/graphene samples; the insets are photographs. Reprinted from [50], with permission from American Chemical Society, 2016.

The coating of an extra layer, such as ZnO [47], aluminum-doped ZnO (AZO) [48] or TiO_2_ [49], by either atomic layer deposition (ALD) or sol-gel methods was found to significantly increase the adhesion. Graphene was also observed to increase the adhesion of Ag nanowires to a polyester yarn surface [50]. This is due to the existence of a large van der Waals force between graphene and the polyester (Figure 5), which helps to bind the Ag nanowires.

Compositing Ag nanowires with substrate polymers is another way to increase the adhesion. Nam and co-workers [51] embedded Ag nanowires in Norland Optical Adhesive (NOA) 63 to obtain strong adhesion. Other materials, such as chitosan [52], alginate [53], and polyvinyl alcohol [54], have also been composited with Ag nanowires, resulting in improved adhesion.

#### 3.2.3. Stretchability

Stretchability is specifically required for Ag nanowire TEs that need to withstand tensile, compressive and shear forces. To meet this requirement, both the Ag nanowire film and the substrate should be stretchable.

Hydroxylated polydimethylsiloxane (PDMS) [55,56,57] and polyurethane urea (PUU) [58] are two of the most commonly used substrate materials for stretchable TEs due to their excellent elasticity. In addition, poly(urethane acrylate) (PUA) [59] and polyimide [44] have also been used for supporting Ag nanowire layers. There are two main routes for depositing Ag nanowires on these substrates. One is to prepare the Ag nanowire film first and to then transfer it onto the substrate [55,56,57]; the other is to fabricate the Ag nanowire-polymer composite first and to then form a film of the composite [59].

The elasticity of the Ag nanowire films is the core of the stretchable TEs and determines the performance of the TEs. Since the Ag nanowires themselves are not elastic, structural designs are needed to ensure the electrical properties of the film under strain.

**Figure 6:**
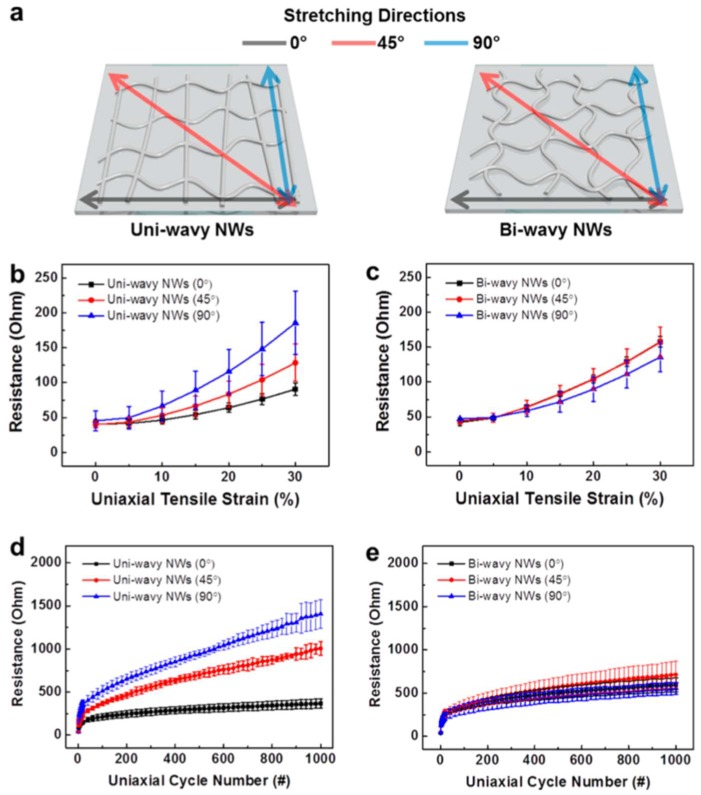
(**a**) Schematics showing the stretching directions of the uniaxial tensile strains for uniwavy NWs and biwavy NWs. Resistance changes as a function of the applied uniaxial tensile strain for (**b**) uniwavy NWs and (**c**) biwavy NWs. Resistance changes as a function of the uniaxial cyclic strain for (**d**) uniwavy NWs and (**e**) biwavy NWs. Electrodes were repeatedly stretched to 30% and released back to 0%. Reprinted from [55], with permission from American Chemical Society, 2017.

A random network of Ag nanowires can lead to increased electrical resistance when multiple cycles of mechanical strain are applied [59]. However, if the Ag nanowires are pre-strained to form a wrinkled structured film (Figure 6), the stretchability of the TEs is significantly enhanced [56]. Another protocol, reported by Kim and co-workers [55], is to make Ag TEs with wavy structured Ag nanowires. This was done by compressing the floating Ag nanowire film before it was transferred onto PDMS.

**Figure 7:**
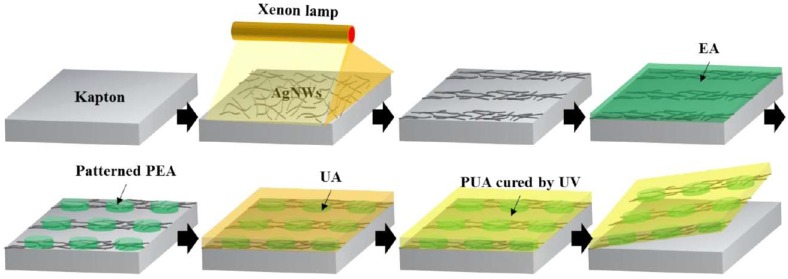
Scheme showing the fabrication of a heterogeneous Ag NW/polymer composite structure. Reprinted from [44], with permission from American Chemical Society, 2017.

In addition to pre-processing the Ag nanowire films, one can also make more complex structures to enhance the performance. Kim and co-workers [44] patterned polyepoxy acrylate (PEA) islands on Ag nanowires deposited on PUA substrates (Figure 7). Using this approach, the stretchability increased significantly, and a 60% strain was sustained for 500 cycles [44].

### 3.3. Chemical Stability

Since silver can react with oxygen and acids, an Ag nanowire-based TE is not chemically stable when it is exposed to air and acids. Additional procedures must be used to enhance the chemicalstability.

The most common way to protect Ag nanowires from chemical reactions is to encapsulate or polymerize the nanowires with a layer of inorganic or organic material. This layer prevents direct contact between the Ag nanowires and potential reactants.

Metal oxides are the most commonly used inorganic materials for the protection of Ag nanowires from oxidation. Atomic layer deposition (ALD) of Al_2_O_3_ onto Ag nanowires was found to effectively protect the nanowires from oxidation [5,60,61]. Alternatively, ZnO [48] and TiO_2_ [43] deposited by ALD were also shown to have the same protective effect. Despite their anti-oxidation effects, some of the metal oxide coatings, such as ZnO and Al_2_O_3_, are not chemically stable in acidic conditions, thereby limiting their application in certain environments.

Glass fabric materials have been used to enhance the chemical stability of Ag nanowire films. A film with a glass-fabric-reinforced transparent composite (GFRHybrimer) embedded with the Ag nanowire networks showed more resistance to corrosive reagents such as 5 wt % K_2_S [62].

Polymers are alternative materials for protecting Ag nanowires through encapsulation [52] or polymerization [63,64]. An epoxy resin protection [65] could extend the lifetime of a silver nanowire TE to 40 days at 85°C and 85% relative humidity. In many cases, the polymers are added for other purposes such as enhancing the adhesion [66]. However, the protective effects are spontaneously added to the Ag nanowires.

Mayousse et al. [34] reported a special case where as synthesized silver nanowires are stable for 2.5 years under lab conditions and for 4 months at 38 °C with 90% relative humidity. However, it is not clear if the nanowires were covered by polymers or organic molecules because the nanowires were synthesized in an organic solution containing polyvinvylprrolidone (PVP) and no washing steps were mentioned in their paper.

### 3.4. Thermal Stability

Thermal stability is required for the application of TEs in high temperature environments. The thermal stability of the polymer substrates is also crucial to TEs, but this issue is not considered in this review. Instead, this section focuses on the thermal stability of the Ag nanowire film.

Ag nanowires are thermally stable at temperatures below 200 °C, while higher temperatures lead to decomposition [67]. The most common phenomenon observed after heating Ag nanowires is that the nanowires shrink upon heating and form droplets. This phenomenon is also elsewhere called negative thermal expansion [68]. It is caused by the coalescence of Ag nanowires [69]. This coalescence is due to the high surface energy of the nanowires and is size [70] and temperature [69] dependent. Smaller nanostructures have higher surface energies [70] and are thus more likely to coalesce, while higher temperatures accelerate the coalescence process [71]. The result of the coalescence is that the nanowires break into small droplets. The origin of the droplet-forming behavior of heated nanowires is called Rayleigh–Plateau instability [14].

**Figure 8:**
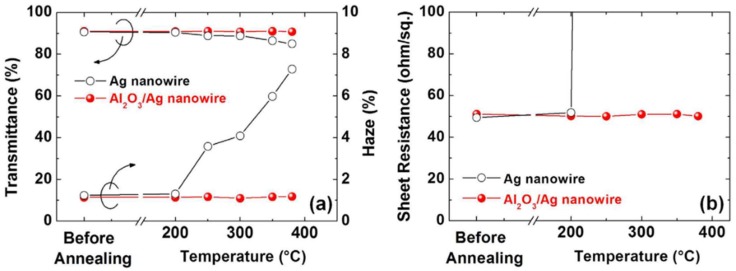
Changes in (**a**) optical transmittance/haze and (**b**) sheet resistance of the Ag and Al_2_O_3_/Ag nanowire electrodes as a function of the annealing temperature. The annealing time is 20 min. Reprinted from [5], with permission from Springer Nature Limited, 2017.

Surface coating with metal oxides is a common way to reduce Rayleigh–Plateau instability and thus enhance the thermal stability. An Ag nanowire film that was coated using the ALD method by ZnO with a thickness of 4.5 nm had long-term thermal stability up to 300 °C [60]. The stability could be improved up to 350 °C by coating with 40 nm thick ZnO [67]. Greater stability was also found for Al_2_O_3_-coated Ag nanowire films (Figure 8), where a 5.3 nm thick Al_2_O_3_ layer increased the stability to 380 °C [5]. TiO_2_ is another material that can be ALD coated on Ag nanowires to enhance the thermal stability [72].

### 3.5. Tuning of the Optical Properties

The total transparency of a TE includes both direct (called specular or clarity) and diffuse transmittance. Direct transmittance refers to the percentage of light that passes through the TE without being scattered. In other words, haze and specular transmittance are the percentage of light that are subject to wide and narrow scattering, respectively. Most studies of TEs focus on the transmittance and the haze. In some applications, high transmittances and low haze are desired, such as in touch panels [14], while in some applications, a high haze is desired, such as in solar cells [73].

The haze of Ag nanowire films depends on the diameter and length of the nanowires. For a single nanowire, the diameter determines the haze. However, for a nanowire film, the lengths of the nanowires are of great importance because the lengths determine the density of the film (also called the area fraction [74] of the nanowires). Therefore, the optical haze can be reduced by using longer and thinner nanowires [54].

**Figure 9:**
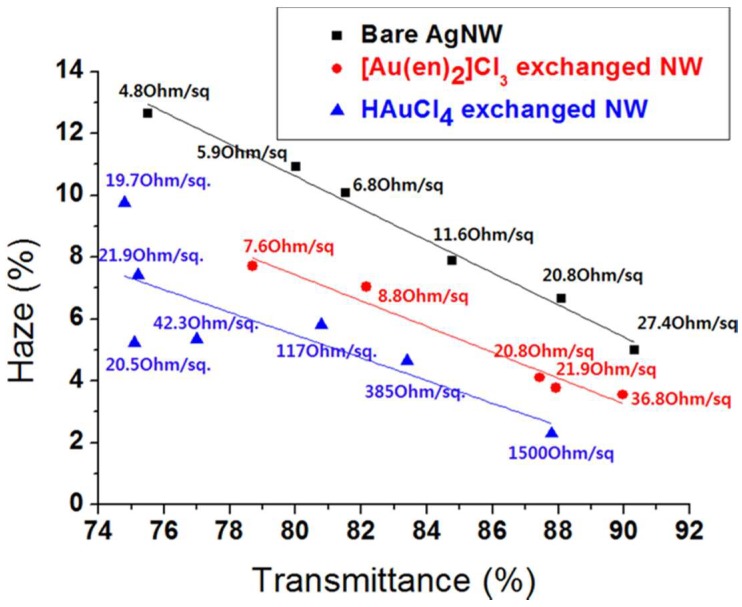
Comparison of haze and transmittance for transparent films fabricated with bare Ag nanowires, Au-coated Ag nanowires made using HAuCl_4_ exchange, and Au-coated Ag nanowires made using [Au(en)_2_]Cl_3_ exchange and NH_3_ treatment. The sheet resistance for each sample is also stated. Reprinted from [77], with permission from American Chemical Society, 2014.

Based on the above, it seems straightforward to make a TE with high transmittance and low haze by using thin nanowires [75]. However, TEs with thin nanowires have lower thermal and chemical stability compared to those with thick nanowires, thereby shortening their lifetime. This requires additional coatings. One route to obtain low haze TEs is to use ultra-long Ag nanowires with relatively thick diameters [76]. Another route to reduce the haze is to coat the Ag nanowires with materials that have smaller extinction coefficients (Figure 9), such as gold [77].

It also seems reasonable to consider making a high haze Ag TE using short and thick nanowires. The problem in this case is that it reduces the transmittance. In practice, researchers use extra coatings, such as FZO, on films made of thin nanowires to increase the optical haze [9].

## 4. Conclusion and Future Perspectives

Silver nanowire-based transparent electrodes (TEs) are promising candidates for replacing transparent conductive oxide (TCO) electrodes. Many studies have shown their excellent mechanical, electrical, optical, and chemical properties, enabling new applications that are not achievable using TCO electrodes. Many protocols have been developed for fabricating Ag nanowire-based TEs with the aim of industrial production.

It is generally recognized that the requirements of industrial production are a transmittance > 90% and a sheet resistance < 100 Ω/sq. Recent advances in making silver nanowire-based TEs have indicated that such requirement could be accomplished easily.

**Figure 10:**
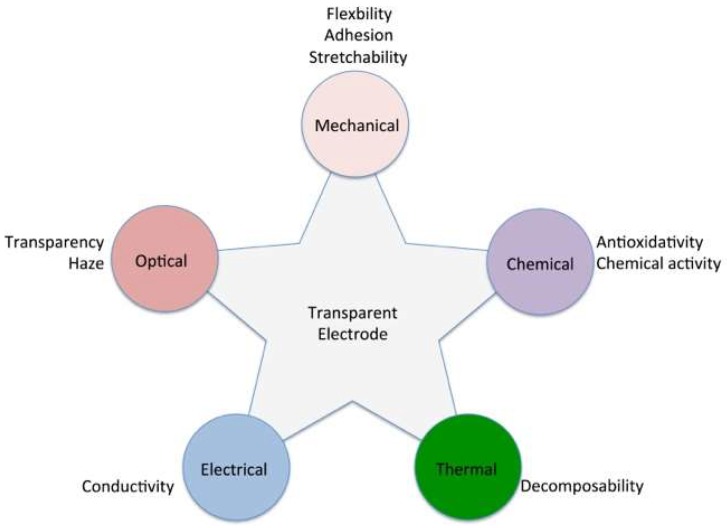
Schematic drawing of the basic factors for evaluating transparent electrodes.

Besides the above-mentioned requirements, there are more factors (Figure 10) to consider than just the requirement of industrial production.

(1) The flexibility of the TEs is becoming increasingly important due to the fast growth of flexible electronics. It is also of great importance for applications of TEs in bio-electronics such as electronic skin. A further requirement of the TEs for biological or biomedical application is the stretchability. Efforts have been made to address this issue, while more studies are needed.
(2) The chemical stability of silver is better than other metals such as copper or nickel that have been used for making TEs. However, TEs made by silver are still sensitive when exposed to air. The current protocol is to cover the silver nanowires with a layer of other materials that can stabilize the TEs. However, deposition of an extra layer such as ZnO usually requires a vacuum process that will limit the production and increase the cost. Improved solutions are expected to solve this problem.
(3) The thermal stability of silver nanowire based TEs has been studied intensively and the results show that the TEs can survive at temperatures higher than 300 °C. Such stability is quite good and can be used in most of the applications.
(4) High transmission of silver nanowire based TEs is aimed for in most of the studies, while few studies focus on the haze property. In most of the applications, high transmission is needed such as display. However, haze is of great importance in applications such as solar cells. There is, on the other hand a lack of methods to tune the transmission and the haze easily to fulfill different requirements.
(5) The conductivity of the silver nanowire based TEs is not a problem as shown by the studies. However, the electrical stability of the TEs has attracted more and more attention because the conductivity of the TEs could reduce over time. Electromigration and Joule heating are the two main reasons. Deposition of an extra layer such as a thin ZnO film could delay the failure although better solutions are expected. Joule heating could be reduced by welding the nanowires to decrease junctions, while simpler methods need to be developed to increase the procedure and reduce the costs.

Recent advances of silver nanowire based TEs have shown their great potential in many applications. This also inspires the future development of small, flexible, and biocompatible electronics that are of great importance for biomedical applications, as well as network communications such as the internet of things (IoT).
